# Hybrid Geopolymeric Foams for the Removal of Metallic Ions from Aqueous Waste Solutions

**DOI:** 10.3390/ma12244091

**Published:** 2019-12-07

**Authors:** Giuseppina Roviello, Elena Chianese, Claudio Ferone, Laura Ricciotti, Valentina Roviello, Raffaele Cioffi, Oreste Tarallo

**Affiliations:** 1 Dipartimento di Ingegneria, Università di Napoli ‘Parthenope’, Centro Direzionale, Isola C4, 80143 Napoli, Italy; claudio.ferone@uniparthenope.it (C.F.); laura.ricciotti@uniparthenope.it (L.R.); raffaele.cioffi@uniparthenope.it (R.C.); 2 INSTM Research Group Napoli Parthenope, National Consortium for Science and Technology of Materials, Via G. Giusti, 9 50121 Firenze, Italy; 3 Dipartimento di Scienze e Tecnologie, Università di Napoli ‘Parthenope’, Centro Direzionale, Isola C4, 80143 Napoli, Italy; 4 Dipartimento di Ingegneria Chimica, dei Materiali e della Produzione Industriale, Università di Napoli Federico II, Piazzale V. Tecchio 80, 80125 Naples, Italy; valentina.roviello@unina.it; 5 Dipartimento di Scienze Chimiche, Università degli Studi di Napoli “Federico II”, Complesso Universitario di Monte S. Angelo, via Cintia, 80126 Napoli, Italy; oreste.tarallo@unina.it

**Keywords:** porous materials, hybrids, geopolymer, adsorption, heavy metals

## Abstract

For the first time, hybrid organic–inorganic geopolymeric foams were successfully used as monolithic adsorbents for the removal of metallic ions pollutants from wastewaters. The foams were realized by the in situ foaming of a hybrid geopolymer obtained by a reaction of metakaolin and polysiloxane oligomers under strong alkaline conditions and then cured at room temperature. In this way, porous materials with densities ranging from 0.4 to 0.7 g/cm^3^ and showing good mechanical properties were produced. With the aim of producing self-standing monolithic adsorbents for the removal of metallic ions pollutants from wastewaters, these porous hybrid geopolymers were subjected to a washing pretreatment with ultrapure water, dried, and then used for absorption tests by dipping them into an aqueous solution with an initial concentration of 20 ppm of Pb^2+^, Cd^2+^, Cu^2+^, and Zn^2+^ ions. Preliminary results indicated that all the tested materials are effective in the adsorption of the tested metal ions and do not release the removed metal ions upon sinking in ultrapure water, even for a very long time. Interestingly, compressive strength tests performed before and after the washing treatments show that the foamed samples remain intact and maintain their physical–mechanical characteristics, suggesting that these kinds of materials are promising candidates for the production of self-standing, monolithic adsorbent substrates that can be easily collected when exhausted, which is a major advantage in comparison with the use of powdered adsorbents. Moreover, since these materials can be obtained by a simple and versatile experimental procedure, they could be easily shaped or directly foamed into precast molds to be used in packed beds as membranes.

## 1. Introduction

Aqueous wastes deriving from different kind of anthropic activities, such as mining or metal industries, contain high concentrations of cations of heavy metals such as Cd, Pb, Cu, Cr, Zi, and Ni. These wastes represent a serious threat to the contamination of soils and waters [1,2,3], as they accumulate in living organisms, generating serious and often lethal pathologies. Therefore, the removal of heavy metal cations from the wastewater of industrial or agricultural activities, before they are released into the environment, is an urgent necessity that has been the subject of studies for several years now. Many effective methods for metals removal from wastewater were developed up to now, including ion exchange, chemical precipitation, electrochemical treatment, reverse osmosis, adsorption, and biosorption [4,5]. In recent years, adsorption has become the central research focus due to its easy approach, effectiveness, and low cost.

Inorganic porous materials are widely studied in the developing of membranes or high-efficiency adsorption materials [6]. In this field, great attention has been devoted to zeolites for their effectiveness in the removal of heavy metal cations from wastewater [7]. Ion exchange properties shown by these materials are due to their unique crystalline microporous structure, characterized by fixed pore dimensions that make them selectively accessible to metal cations, depending on their charge and size [8,9].

Geopolymers are inorganic materials, similar to zeolites, but showing an amorphous three-dimensional aluminosilicate network structure. Unlike zeolites, these materials are characterized by simple and cost-effective synthesis procedures [10] based on the reaction of natural Si- and Al-rich materials, such as metakaolin or industrial by-products (fly ash, blast furnace slag, muds) in highly alkaline aqueous solutions. A polycondensation reaction leads to the formation of a gel network consisting of SiO_4_ and AlO_4_ tetrahedra sharing oxygen corners and forming rings of various sizes, which are analogous to those found in zeolites. 

Geopolymers exhibit a wide variety of technologically relevant properties, such as high compressive strength [11], fire resistance, and low shrinkage [12]. For these reasons, geopolymers seem to be a valuable alternative to ordinary Portland cement, which is also thanks to their environmentally sustainable fingerprint [13,14,15] being mainly associated with the reduced CO_2_ emissions characterizing the raw materials from which they can be obtained [16]. When foaming agents (as hydrogen peroxide or metallic Al or Si powders) are added into the geopolymer paste during its consolidation, porous materials with pore sizes ranging from a few tenths of nanometers to a few millimeters can be obtained [17,18,19]. At variance with conventional techniques used for the production of porous ceramics [20], no high-temperature treatments (such as burn out of organics and sintering) are necessary for this process. 

Many studies report on the capability of geopolymers to remove heavy metal ions from aqueous solutions [21,22,23,24,25,26,27,28,29]. These studies are all related to metakaolin or fly ash-based geopolymers. However, when immersed in water, in some cases, the formation of efflorescence has been found, while for samples characterized by elevated Na_2_O/Al_2_O_3_ ratios (>1.5), the disintegration of the specimen has been experienced [30]. Moreover, in order to maximize the surface area of the adsorbent, these materials are not used as large monolithic artifacts; instead, they usually are finely ground and reduced to a fine powder which is then further sieved [31].

Recently, we succeeded in synthetizing geopolymer-based organic–inorganic composites and hybrid materials by reacting an aluminosilicate source and an aqueous alkali hydroxide and/or alkalisilicate solution with mixtures of dialkylsiloxane oligomers or organic resins precursors [32,33]. With respect to the unmodified geopolymers with an analogous Si/Al ratio, these materials are characterized by enhanced mechanical properties, along with good temperature and fire resistance [34,35,36,37,38,39,40]. By adding a foaming agent, these materials have been obtained as foams with densities ranging between 0.2 and 0.8 g/cm^3^ and showing good mechanical properties and low thermal conductivity, which makes them particularly suitable for the utilization as insulating materials in the field of constructions [39,40,41].

To date, to the best of our knowledge, no studies have been reported on the absorption capability of geopolymer-based organic–inorganic hybrid foams. This last class of materials could be of particular interest since, due to its superior mechanical resistance with respect to unmodified geopolymers with analogous chemical composition [33], it could be effectively used as adsorbent for the production of self-standing large monolithic absorbent substrates, with no need to be previously ground. In fact, they could combine the well-known adsorbent capacity of geopolymers with the improved mechanical performance characterizing geopolymeric hybrid foams [39], which would make them more suitable for practical applications in aqueous environments than the unmodified geopolymers. As a matter of fact, geopolymeric hybrid foams combine (i) a tunable porosity and morphology with versatile rheological properties that make them easy to mold; (ii) good mechanical properties after the consolidation, which makes them useful for the production of reusable large filters or membranes that are capable of being recovered intact after use; and (iii) a simple, environmentally friendly, and cheap process of production [33,39].

In this paper, for the first time, the capability of these hybrid organic–inorganic geopolymeric foams to act as sieves for removing heavy metal cations from aqueous solutions in the form of self-standing large substrates has been studied. The collected results indicate that these hybrid materials are effective in the adsorption of several metallic species, such as Zn^2+^, Cd^2+^, Pb^2+^, and Cu^2+^ ions, which can be acutely toxic for humans and other organisms at elevated concentrations, whilst a prolonged exposure to lower concentrations can cause serious diseases [42,43,44]. Moreover, these new foams do not release the removed metal ions when placed again in ultrapure water, even for a very long time. Finally, compressive strength tests performed before and after the washing treatments show that the foamed samples remain intact and maintain their physical–mechanical characteristics, suggesting that they could be effectively used as an adsorbent for the production of self-standing large absorbent substrates, which could be easily shaped or directly foamed into precast molds.

## 2. Materials and Method

### 2.1. Materials

The metakaolin that was used in this paper as a raw material was provided by Neuchem S.r.l. (Milan, Italy), while sodium silicate solution was supplied by Prochin Italia S.r.l (Caserta, Italy). Table 1 reports their chemical composition. Reagent grade sodium hydroxide and silicon powder (~325 mesh) were supplied by Sigma-Aldrich (St. Louis, MO, USA). A commercial oligomeric dimethylsiloxane mixture, Globasil AL20, was purchased from Globalchimica S.r.l (Turin, Italy).

Single component standard solutions of lead, copper, zinc and cadmium cations (in concentration of 1000 ppb) and multistandard solutions of anions and cations were obtained from Honeywell Fluka^TM^ (Bucharest, Romania).

### 2.2. Preparation of Hybrid Geopolymer Foams (GSil)

Hybrid polysiloxane–geopolymer slurries were prepared as described elsewhere [33,39]. In order to obtain a set of samples characterized by a different degree of porosity, silicon powder was added to two distinct aliquots of the hybrid suspensions in 0.03 wt% and 0.12 wt% ratios, and then the whole system was mixed for a further 5 min at 1000 rpm. The obtained foamed samples, once consolidated after curing treatments (see below), are hereafter indicated as GSil03 and GSil12, respectively. The relevant details of the mix design are reported in Table 2.

As soon as prepared, all the foaming slurries were casted into cubic molds and cured in >95% relative humidity conditions at ≈22 °C for one day and then at 60 °C for further 24 h. Afterwards, the samples were kept at the same temperature in >95% relative humidity conditions for a further 5 days and finally for a further 21 days in air. 

In order to perform microstructural and mechanical studies and evaluate the adsorption capability with respect to different metal ions, for each composition, samples of different dimensions and shapes were prepared. In particular, adsorption tests were performed on platelets obtained by cutting each sample into 2 × 20 × 20 mm^3^ and 5 × 20 × 20 mm^3^ slices: the samples with a thickness equal to 2 mm were named samples “A” (GSil03A, GSil12A), while those 5 mm thick were named samples “B” (GSil03B, GSil12B). The dimensions of the specimens were chosen in order to fit the in-house developed specimen holders used for adsorption and desorption tests. The apparent density and mass of the samples used for the adsorption tests are reported in Table 2. Before performing the adsorption tests, the obtained foamed platelets were subjected to a preconditioning washing step, as will be described in the following sections.

### 2.3. Methods

#### 2.3.1. Physical and Microstructural Assessment

SEM analyses were carried out by means of a Nova NanoSem 450 FEI Microscope. Microcomputed tomography (µCT) was performed at the ATeN Center (University of Palermo, Italy) with a µCT scanner (Skyscan 1272, Bruker, Kontich, Belgium) equipped with a 1 mm aluminum filter. The samples were scanned at 40 kV source voltage and 250 mA current. A rotation of 180° by 0.2° steps was carried out. Pixel size was 7.4 µm. Two-dimensional (2D) reconstructions were carried out using the NRecon software (version 1.6.10.2, Allentown, PA, USA) with 8-bit color depth and 265 gray level. After that, the whole set of raw images were displayed in 3D by CTVox software. Quantitative analyses were carried out via CTan software (version 1.16.1.0). These measurements allowed also evaluating the porosity of the samples.

Apparent density evaluations were carried out by means of an OHAUS-PA213 hydrostatic balance provided by Pioneer.

#### 2.3.2. Compressive Behavior

Uniaxial compressive tests were carried out as already reported in ref. [39] on 50 × 50 × 50 mm^3^ cubic specimens. Two sets of samples were examined: the first set of measurements were performed on “as-prepared” specimens, i.e., on samples as obtained after the curing stage, before the washing treatments; the second set of measurements was performed on the specimens after having carried out the washing procedure described in Section 2.3.3 on them and until the ion release measurements and the pH of the washing solutions were the same as those recorded for the specimens subjected to the adsorption tests described in Section 3.2. For each sample type, at least three specimens were tested under displacement control in order to obtain the corresponding stress–strain curve, compressive strength, and Young’s modulus, and the values reported are the averages of the values obtained. More experimental details are reported in ref. [39].

#### 2.3.3. Washing Procedure and Adsorption/Desorption Tests

Hybrid geopolymer foams were carefully washed to clean their surface and internal pores from residues due to the synthesis process and cutting step. The washing procedure was also necessary to avoid contamination of the aqueous solution during metals adsorption tests and to control the effects of the artifacts on the alkalinity of the aqueous solution. In fact, it was observed that a metakaolin-based geopolymer can determine an increase of the pH of aqueous solution, thus conditioning metal’s precipitation equilibrium [27].

To this aim, each sample was dipped in 50 mL of ultrapure water for 7 days. Periodically, the bath was manually shaken. After this time, aqueous solutions were recovered, filtered with filters with pores of 0.2 µm, and frozen until analyses were performed on them. The process was repeated four times, and chemical analyses were carried out on each washing solution obtained (see the next paragraph). After washing steps, samples were dried in an oven at 50 °C for 24 h until constant mass and then tested for their adsorption capacity.

Adsorption tests were performed on each hybrid foam by dipping the platelets in a bath containing 50 mL of an aqueous solution of each metal cation (Pb^2+^, Cd^2+^, Cu^2+^, Zn^2+^) with an initial concentration of 20 ppm. These solutions were obtained by diluting single metal standard solutions at a starting concentration of 1000 ppm.

Each foam was kept in contact with the solution for 7 days; after this time, solutions were recovered, filtered with filters with pores of 0.2 µm, and then frozen until analyzed.

Metal cations desorption tests were performed as follows. Each hybrid foam used for absorption tests was kept for 7 days in a bath with 50 mL of ultrapure water at room temperature (24 °C) without agitation. After this contact time, solution baths were recovered and analyzed for their metal cations content.

#### 2.3.4. Chemical Analyses of Aqueous Solutions from Washing Steps: Absorption and Desorption Tests

Aqueous solutions from washing steps were analyzed for their content of major ions and some metal cations (Pb^2+^, Cd^2+^, Cu^2+^, Zn^2+^), while solutions from absorption and desorption tests were analyzed for their metal cations content only.

For the major ions, determination ionic chromatography was performed with a Dionex 1100 system, which was designed for the contemporary determinations of anions and cations; specifically, anions (Cl^−^, F^−^, Br^−^, NO_2_^−^, NO_3_^−^, PO_4_^3−^, and SO_4_^2−^ as inorganic species, and HCOO^−^, CH_3_COO^−^, and C_2_O_4_^−2^ as organic species) were determined using a line constituted by an ASRS 300–4 mm suppressor with an applied current of 33 mA, an AS22 column working with a cell volume of 100 μL, and a buffer solution with title of 3.5 mM of sodium carbonate/bicarbonate as eluent (flow rate 1.20 mL/min). The line for cations (Li^+^, Na^+^, K^+^, NH_4_^+^, Ca^2+^, and Mg^2+^) determination was constituted by a CERS 500–4 mm suppressor with an applied current of 15 mA, a CS12A column working with a cell volume of 25 μL, and 20 mM methanesulfonic acid solution as the eluent (flow rate 0.25 mL/min); for both anions and cations, calibration curves were defined using certified multistandard solutions.

The voltammetric analyses for metals determination were carried out with a Metrohm 797 VA Computrace equipped with a multimode working Hg electrode. An Ag/AgCl electrode was used as the reference, and a Pt electrode as the auxiliary electrode. The standard addition method was applied as the calibration method to limit the matrix effects. All elements were quantified using linear regression based on the height of the peaks of the voltammograms. This method allows the determination of zinc, cadmium, lead, and copper. The pH of the solutions was determined with a Metrohm 781 pH/Ion meter. The compliance and reliability of analytical procedures were discussed in ref. [45].

The percentage removal efficiency (*E*) and efficiency for mass unit of geopolymer (*Q*) were evaluated as follows:

(1) $$E\;(\%)=\frac{C_{o}-C_{e}}{C_{o}}\times 100$$

(2) $$Q=\frac{\left(C_{o}-C_{e}\right)V}{W}$$

where *C_o_* was the initial metal concentration (as mg/L), *C_e_* was the final metal concentration after the adsorption test (as mg/L), *V* was the volume of solution (as L), and *W* was sample weight (as g).

## 3. Result and Discussion

### 3.1. Porous Hybrid Geopolymeric Materials: Preparation and Microstructural Characterization

As stated before, in this paper, hybrid organic–inorganic geopolymeric foams were successfully used as monolithic adsorbents for the removal of metallic ions pollutants from wastewaters. To this aim, two sets of foamed hybrid samples with different porosity, GSil03 and GSil12, were prepared. In particular, in order to evaluate the adsorption capability of these materials as monolithic adsorbents, rectangular specimens of different dimensions and thicknesses (Table 2) were shaped. The advantage in the obtainment and use of hybrid geopolymer-based materials with respect to unmodified geopolymers relies in the fact that the homogeneous combination of inorganic and organic moieties in a single-phase material provides unique possibilities to obtain properties that are not found in the organic polymer or in the inorganic materials independently. In particular, the produced hybrid foams show significantly increased mechanical properties [33] with respect to the unmodified geopolymer reported in the literature [46,47,48,49,50].

The hybrid foams were prepared by applying a synthetic approach earlier developed by some of us [33] consisting of the simultaneous polymerization of a commercial oligomeric polydimethylsiloxane mixture with metakaolin (MK) under strong alkaline conditions. During the polycondensation reaction, the formation of chemical bonds between the reactive MK-based geopolymeric suspension and the polydimethylsiloxane mixtures takes place, thus allowing the obtainment of a hybrid material [33]. It is worth emphasizing that the choice of metakaolin as the aluminosilicate source has been driven by the consideration that this material is purer and more homogeneous in its chemical composition than the industrial by-products [51] that are commonly used as raw materials. For this reason, metakaolin is more suitable than ash or muds for the obtainment of high value-added artifacts, such as those proposed in the present paper.

In order to obtain monolithic foams characterized by different porosity, Si^0^ powder was added as the blowing agent into the freshly prepared slurry: the formation of voids takes place due to the evolution of hydrogen gas during the reaction of silicon with water molecules in alkaline environment. Obviously, the foaming process was strongly dependent on the amount of Si^0^ added, and as expected, the volume expansion of the slurry increased by increasing the amount of the foaming agent; on the contrary, the density of the cured porous materials decreases as the amount of foaming agent increases (Table 2).

**Figure 1:**
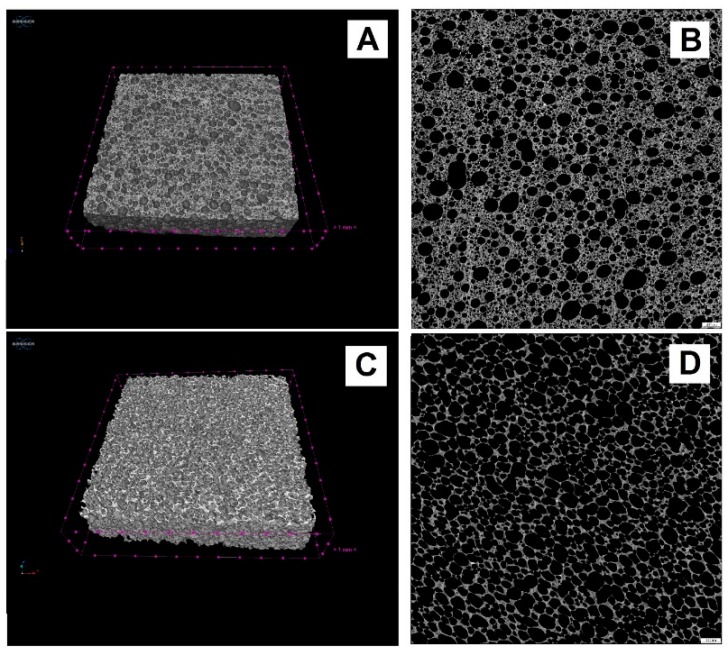
X-ray microtomography images of 3D slice (on the left) and 2D slice (on the right) of the GSil03 sample (**A**,**B**) and of the GSil12 sample (**C**,**D**). The scale bar is 500 μm.

**Figure 2:**
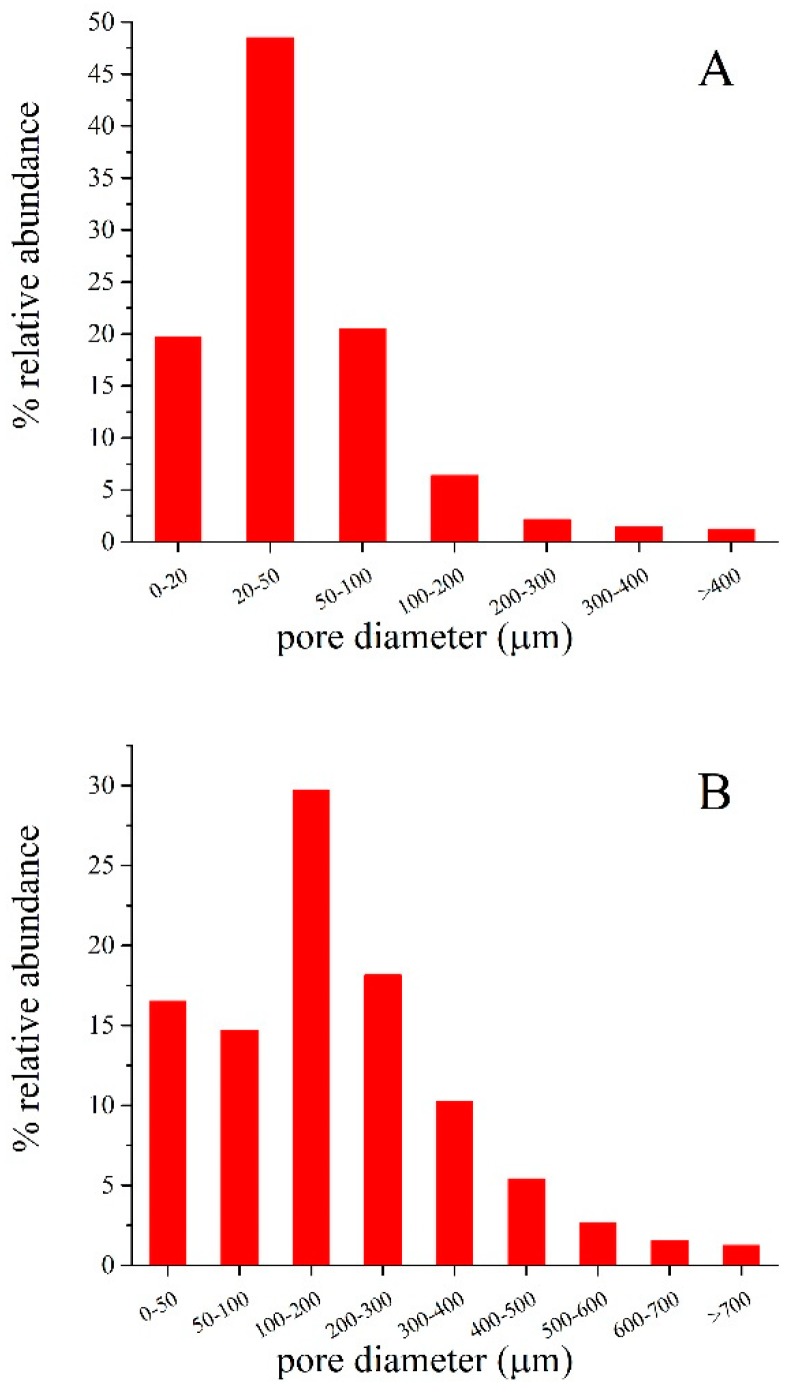
Pore size distribution in GSil03 (**A**) and GSil12 (**B**) as obtained by analysis of the microtomographies reported in Figure 1.

The morphology of the foamed specimens obtained has been studied in detail by means of X-ray microtomography and SEM. Figure 1 shows 2D and 3D X-ray microtomography images of (1 × 1 × 1 cm^3^) slices of GSil03 and GSil12 samples. These images show the presence of pores of different size and shape that, in both cases, are homogenously distributed within the sample. It is worth noting that the highest Si^0^ addition as the foaming agent performed in GSil12 (Figure 1C,D) produces a foam of lower density: in particular, by processing 2D and 3D microtomography, it resulted that the total porosity of the samples GSil03 was ≈50%, while that of GSil12 foam was ≈72%. This porosity turned out to be constituted almost exclusively by open porosity (≈46% for GSil03 and ≈71.7% for GSil12). As far as the shape of the pores, GSil03 presented almost perfectly circular pores, with a minor anisotropy along the main foaming direction (Figure 1A,B). Instead, the GSil12 sample is characterized by a less regular shape of pores, which is probably due to coalescence phenomena (Figure 1C,D). Moreover, as shown by the pore distribution analysis reported in Figure 2a, 90% of the pores of the GSil03 sample had a size between a few microns up to 100 μm, ~6% of the pores had a size included in the range 100–200 µm, and only ~4% of the pores were over 200 µm. Instead, in the case of GSil12 (Figure 2b), the percentage of pore diameters up to 100 µm is only ~31%, while most of the pores (~58%) have a diameter in the range 200–400 µm. Meanwhile, 11% of pores in this sample present a diameter larger than 400 µm. Of course, this very different pore distribution in the two foams and the fact that GSil12 foam presents the majority of pores larger than 100 µm is due to the coalescence of the gas bubbles due to the use of the highest quantity of foaming agent.

**Figure 3:**
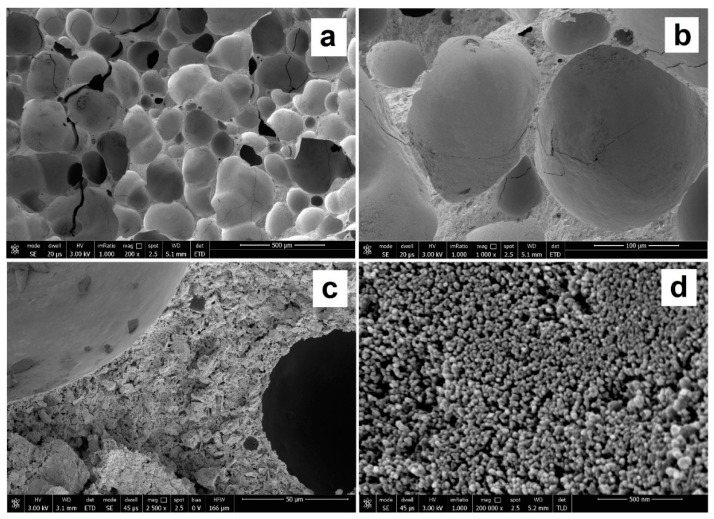
SEM micrographs at 200 (**a**), 1000 (**b**), 2500 (**c**), and 200,000 (**d**) magnifications of freshly obtained fractured surfaces of GSil03 foams after washing pretreatment (see Section 3.2).

In order to get a better insight of the microstructure of the samples prepared, SEM micrographs of freshly obtained fractured surfaces of the studied foams were performed. Figure 3 shows SEM images of the GSil03 sample after the washing pretreatments, as it was used for the adsorption tests (see Section 3.3). The results of the SEM investigations support the findings of the microtomography, but they also additionally provide insight into the structure of the walls between the pores and of the pore surface itself. In particular, this sample shows a quite regular distribution of the pores due to the evolution of hydrogen gas during the foaming process (see Figure 3a,b). A deeper inspection of pore walls (Figure 3c) points out that the sample exhibits a continuous and uniform structure in which no segregation phenomena are observable, even at the nanometric scale level, although it contains an organic component. This is attributable to the formation of a tridimensional and interpenetrated network in which the inorganic (geopolymeric) phase and the polysiloxane component are able to strongly interact by means of the formation of chemical bonds between the aluminosilicate and organo-siloxane units. Within pores, the presence of a nodular morphology reminiscent of the gel formation in the early stages of geopolymeritazion, with an average diameter of nodules of about 40–50 nm, is also evident (Figure 3d) [33].

**Figure 4:**
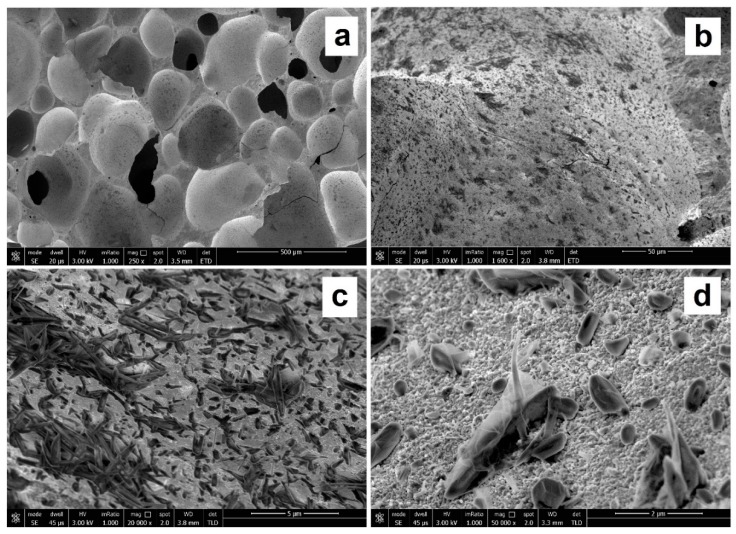
SEM micrographs at 250 (**a**), 1600 (**b**), 20,000 (**c**), and 50,000 (**d**) magnifications of freshly obtained fractured surfaces of GSil03 foam before washing pretreatment (see Section 3.2).

Figure 4 shows the SEM micrographs of the GSil03 sample already examined in Figure 3, but taken before the washing treatment of the sample. These images revealed that the presence of deposits on the surface of the “as-prepared” sample is likely due to salts and microcrystals, which were effectively removed through the washing treatment carried out before the adsorption tests (Figure 3). As will be discussed in Section 3.2, besides freeing the surface from these residues, the washing pretreatment we performed before the adsorption tests removed ions and salts deriving from raw materials and from the geopolymerization reaction, thus making the foamed samples usable as adsorbent materials.

### 3.2. Chemical Composition of Washing Solutions

**Figure 5:**
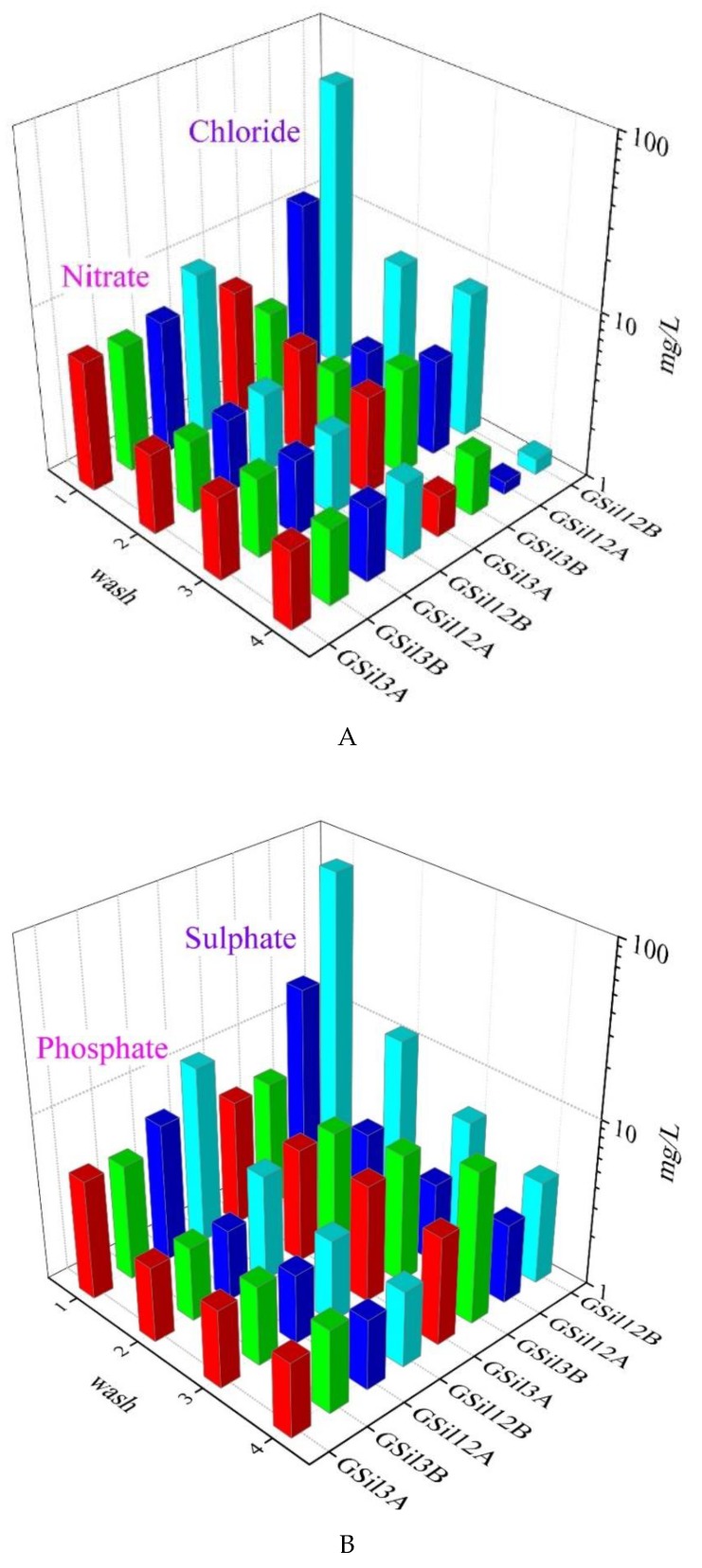

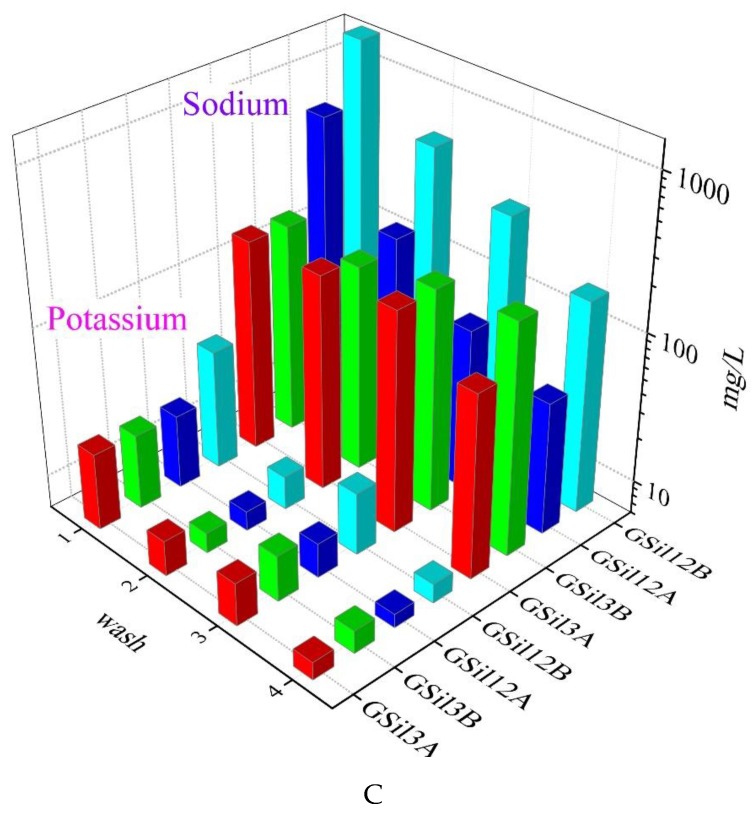
Major ions concentration (nitrate, chloride (**A**); phosphate, sulfate (**B**); potassium, sodium (**C**) expressed as mg/L, collected after each washing step of GSil03 and GSil12 foams.

The concentration of fluoride, chloride, nitrate, phosphate, sulfate, sodium, and potassium ions in the washing solutions as obtained after each washing cycle are reported in Figure 5. Other common ions, such as acetate, formiate, oxalate, bromide, nitrite, calcium, magnesium, and ammonium, were not detected.

As expected, in the first washing solution, the most abundant ion was sodium, since it was contained in the alkaline activating solution used in the polycondensation reaction. In particular, the concentration of Na^+^ in the first washing cycle was up to 160 mg/L for GSil03 samples and up to 1550 mg/L for GSil12 samples (Figure 5C). The great difference in these values could be related to the fact that GSil12 samples presented a greater surface area with respect to GSil03 being characterized by a greater porosity, as discussed in the previous paragraph. Moreover, it is worth pointing out that within each set of samples (GSil03A and GSil03B; GSil12A and GSil12B), the high difference in the release of cations (for example, the GSil12B sample released 2.5 times more Na^+^ ions than GSil12A) could be explained considering again that the “B” samples have a greater surface area of “A” samples, being thicker (see Table 2). This hypothesis may be supported by the fact that if we normalize the concentrations in washing solutions of the monitored ions by the mass of the used geopolymer foams, the differences previously observed decrease considerably (Table 3). This observation was in line with our expectations, since the chemical composition of the samples was the same, and since they differed only for the porosity.

All the other monitored ions (K^+^, F^−^, Cl^−^, NO_3_^−^, PO_4_^3−^, SO_4_^2−^) were detected at much lower concentrations (Figure 5). Fluoride ion (not reported in Figure 5) was the less abundant species being practically absent in the washing solutions of all the specimens examined (0.000 ± 0.002 mg/L for the GSil03A sample, 0.018 ± 0.006 mg/L for the GSil03B sample, 0.896 ± 0.022 mg/L for the GSil12A sample, and 4.624 ± 0.032 mg/L for the GSil12B sample).

The concentrations of all species, with the exception of sodium for the GSil3B sample, decreased proceeding with the washing cycle and, after the fourth washing step, the concentration of chloride, fluoride, sulfate, nitrate, and phosphate were well below limits imposed by the Italian Standard law for waste water and defined by the Law Decree 152/2006.

In our opinion, this experimental observation is an indirect confirmation of the effectiveness of our conditioning method that is able to remove ions even for massive foamed samples (as GSil12B), as long as the porosity of the specimens is mostly open porosity.

**Figure 6:**
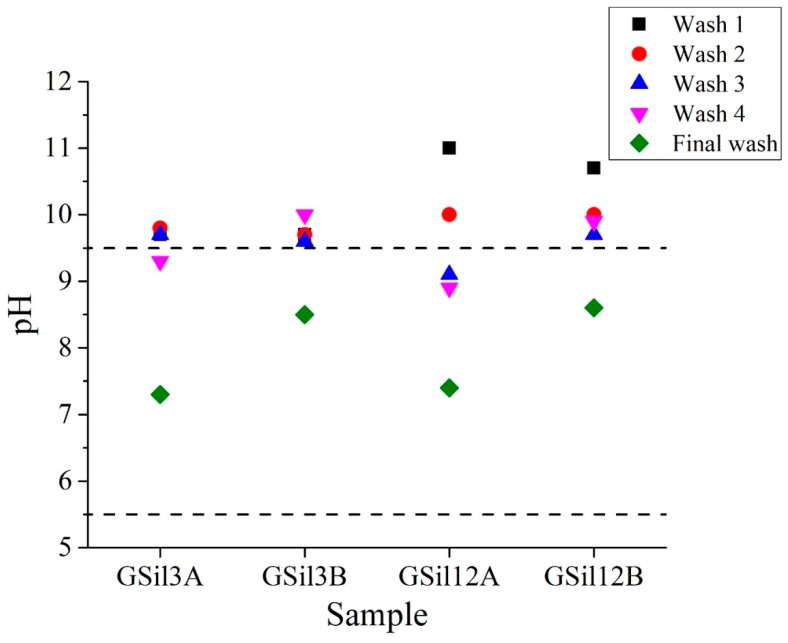
pH of washing solutions as determined soon after the first (black squares), second (red circles), third (blue triangles), and fourth (magenta triangles) washing cycle and after the final wash with a current of ultrapure water (green diamonds). Dotted lines delimit the pH range of Italian standard law.

As far as the pH of washing solutions (Figure 6), it is worth noting that despite the fact that four washing cycles have been able to effectively reduce the major ions concentration, this kind of conditioning treatment was not enough to bring the pH values within the limits imposed by Italian law (i.e., in the range 5.5–9.5). In particular, after four washing cycles, the pH decreased below limits just for GSil03A and GSil12A (that is, the thinner samples), while both thicker samples (GSil03B and GSil12B) retained high pH values, which was neither compliant with the limits imposed by law nor for adsorption tests conditions. For this reason, the samples were subjected to a final wash with a current of ultrapure water until the pH reached acceptable values (in the range of 7.3–8.6, green diamonds in Figure 6).

Finally, washing solutions were also analyzed for their content of Pb^2+^, Cd^2+^, Cu^2+^, and Zn^2+^. The detected concentrations of metal cations were always well below limit of detection, thus meaning that overruns of standard limits (fixed by Italian Law Decree 152/2006 and corresponding to 0.002 mg/L, 0.2 mg/L, 0.1 mg/L and 0.5 mg/L for cadmium, lead, copper and zinc respectively) were not observed. For these reasons, no solution contamination was observed, thus allowing performing adsorption tests.

### 3.3. Adsorption and Desorption Tests: Preliminary Results

**Figure 7:**
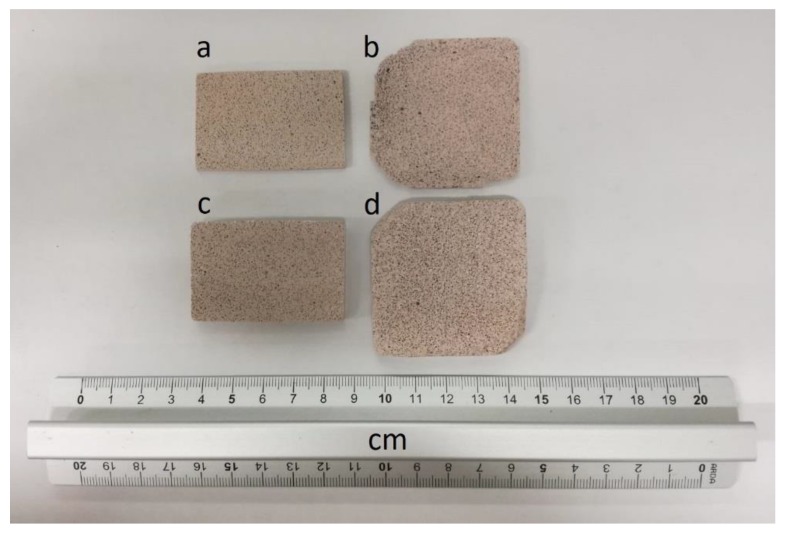
Optical image of GSil03 (**a**,**b**) and GSil 12 (**c**,**d**) hybrid foams as recovered after the adsorption and desorption tests. The a,c samples are 2 mm thick, while the b,d samples are 5 mm thick.

After washing treatments, the conditioned foamed samples were dried and then used for absorption tests by dipping them into an aqueous solution with an initial concentration of 20 ppm of each metal cation (Pb^2+^, Cd^2+^, Cu^2+^, Zn^2+^). Figure 7 show an optical photograph of the specimens used for the adsorption tests. It is worth noting that despite the prolonged water treatments that have been performed, no efflorescence has formed on the surface of the samples.

Metal ions concentrations in the solutions after the adsorption test were reported in Table 4. It is apparent that all the tested hybrid foams strongly knock down the metal cations concentration with percentage efficiency values (E) in the range of 99–100%. No significant differences among hybrid foams due to thickness or porosity could be pointed out, neither among the different metal ions tested.

With the aim of better quantifying the capability of the foam with respect to metals adsorption, the efficiency for the mass unit of geopolymer foam (*Q*) was also evaluated. This parameter resulted in a range of 0.1–0.4 (Table 4). It is important to note that these values refer to a specific test that has been performed using a relatively high adsorbent concentration (55 g/L, 173 g/L, 63 g/L, and 191 g/L for GSil 3A, GSil 3B, GSil 12A, and GSil 12B, respectively) in a solution with a low metal ions concentration (20 mg/L), so they do not represent the maximum metal amount that this kind of hybrid geopolymer foam could adsorb, but they point out that the studied material is able to effectively remove metal cations from aqueous solutions even if shaped in relatively big monolithic artifacts (see Figure 7) and not as fine powders, as usually done in the literature for geopolymer-based materials [23]. It is interesting to note that *Q* was highest for GSil3A and GSil12A foams—that is, for samples with smaller thickness—probably because their smaller thickness made it possible for the cations to easily reach even the most internal sites of the specimens.

Finally, since the adsorption capability of metal ions was tested simultaneously, we have also evaluated a cumulative adsorption efficiency, *Q_tot_*, according to Equation (2) where *C_o_* was the initial total metal concentration (as mg/L), and *C_e_* was the final total metal concentration after the adsorption test (as mg/L). *Q_tot_* was found to be equal to 1.533 for GSil3A, 1.365 for GSil12A, 0.480 for GSil3B, and 0.440 for GSil12B.

Hybrid foams were also tested for their capability of retaining the metal cations absorbed from the solution in the adsorption test described before. In general, the desorption test pointed out that the studied artifacts released very low concentrations of metal ions, in many cases below instrumental detection limits, without significant differences among metals and the different geopolymer samples tested (Table 4).

This indicated that metal ions were somehow linked to the hybrid geopolymer and were efficiently retained.

### 3.4. Mechanical Properties

As pointed out before, differently from what is usually done in the literature where analogous geopolymeric materials are used as fine powders [31], in order to evaluate the possibility of realizing self-standing monolithic filters based on porous geopolymer hybrids, all the adsorption and desorption tests were performed on monoliths. As shown in Figure 7, after the adsorption and desorption tests, all the tested samples were recovered without cracks. 

**Figure 8:**
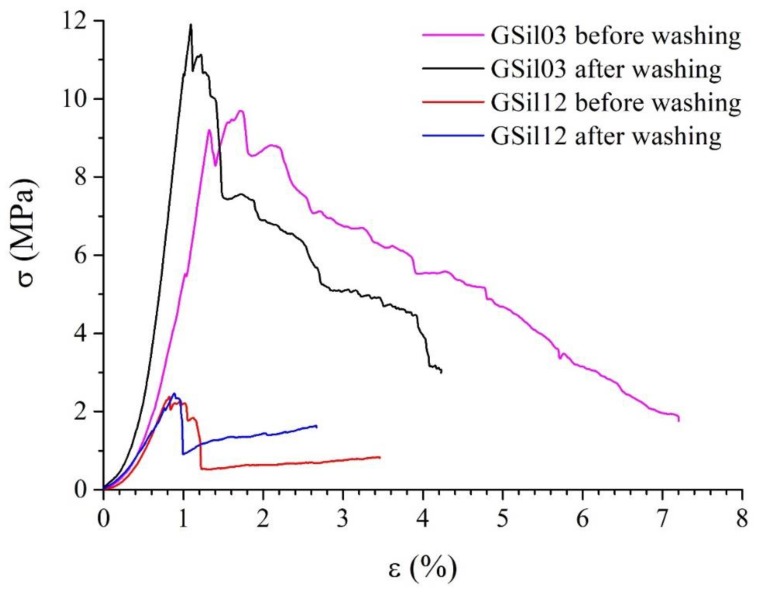
Stress–strain curves in compression of GSil03 and GSil12 samples before and after the washing procedure. See text for details.

For this reason, aiming at verifying the mechanical properties of the artifact after their immersion in water for a long period of time, compressive strength tests on 50 × 50 × 50 mm^3^ cubic samples were performed according to ASTM D1621 (more details can be found in reference [33]) before and after washing treatments according the same procedure described in Section 2.3.3. In particular, also on these cubic specimens, the washing conditioning was performed until the ion concentration and the pH of the washing solutions were in agreement with the results obtained for the specimens subjected to the adsorption tests (Section 3.2). Figure 8 shows the stress–strain curves obtained. It is manifest that the mechanical resistance of the cubic specimens tested after their washing was completely comparable with that of the specimens before washing. In particular, for each foam, the stress–strain curves recorded before and after the washing treatment were found to be very similar (Figure 8). Both foams exhibited a well-defined elastic regime, which was noticeable at the early stages of stress. The linear elastic regime remained with an almost constant slope until reaching the yield or unstable collapse point, which was characterized by a sharp load loss for GSil12 and by a less drastic behavior for GSil03. The average values of compressive strength (calculated from the maximum load applied to the specimens and corresponding to the yield or unstable collapse point) and Young’s modulus under compression (derived from the slope of the initial linear region of the stress–strain diagram) are reported in Table 5.

To date, to the best of our knowledge, good mechanical properties combined with interesting adsorption capacity have been reported in the literature only if zeolitic tuff is used as a fine aggregate in geopolymeric binders [52,53].

It is worthwhile to point out that the new hybrid material we propose combines good mechanical properties with the capacity to absorb pollutants, as advocated by several authors in the literature, who have recently advanced for other materials based on geopolymers, the possibility to use them also as non-structural construction materials for water tanks, pipes, and damns [54,55].

## 4. Conclusions

In this work, hybrid foams realized by the reaction of metakaolin and polysiloxane oligomers have been tested for their efficiency as non-conventional adsorption media for metallic pollutants with the aim of producing self-standing monolithic absorbent substrates. Preliminary results indicated that the studied materials are effective in the adsorption of Zn^2+^, Cd^2+^, Pb^2+^, and Cu^2+^ and they do not release the removed metal ions upon dipping in ultrapure water, even for a very long time. Moreover, we demonstrated that the monolithic artifacts used in this study remain intact and were recovered without cracks after washing treatments and adsorption and desorption tests, thus maintaining good mechanical properties. At variance with the analogous adsorbing materials based on geopolymers described in the literature up to now, which are usually finely ground and reduced to a fine powder, the good mechanical properties of these hybrid materials suggest their application as promising candidates for the production of large, self-standing adsorbent substrates. These substrates could be even easily shaped or directly foamed into precast molds and easily collected when exhausted, which are major advantages over powdered adsorbents.

## Figures and Tables

**Table 1:** Chemical composition (weight %) in terms of major oxides of the metakaolin and the sodium silicate solution used to obtain the specimens tested in this paper.

**Metakaolin**							
SiO_2_	Al_2_O_3_	TiO_2_	Fe_2_O_3_	K_2_O	MgO	CaO	others
52.90	41.90	1.80	1.60	0.77	0.19	0.17	0.67
**Sodium Silicate Solution**							
SiO_2_	Na_2_O	H_2_O					
27.40	8.15	64.45					

**Table 2:** Mix composition (wt%), apparent density, and mass of the studied samples. MK = metakaolin; SS = sodium silicate solution; DMS = oligomeric dimethylsiloxane mixture; Si = silicon powder.

Sample	MK	SS	NaOH	DMS	Si	Apparent Density (g cm^−3^)	Sample’s Mass * (g)
GSil03	37.4	45.0	7.6	10.0	0.03	0.701 ± 0.002	2.60990 ± 0.00001 (A) 8.33520 ± 0.00001 (B)
GSil12	37.4	45.0	7.6	10.0	0.12	0.396 ± 0.003	2.93095 ± 0.00001 (A) 9.02261 ± 0.00001 (B)

* Mass of the samples used for the adsorption tests after washing treatment and drying.

**Table 3:** Major ions concentration in washing solutions as determined after each washing cycle of the specimens, expressed as (mg of ion)/(g of hybrid geopolymer foam).

Sample	F^−^ (mg/g of Adsorbent)	Cl^−^ (mg/g of Adsorbent)	NO_3_^−^ (mg/g of Adsorbent)	PO_4_^3−^ (mg/g of Adsorbent)	SO_4_^2−^ (mg/g of Adsorbent)	Na^+^ (mg/g of Adsorbent)	K^+^ (mg/g of Adsorbent)
After wash cycle No. 1							
GSil3A	0.000	0.104	0.106	0.091	0.100	2.777	0.342
GSil3B	0.000	0.019	0.033	0.028	0.033	0.860	0.106
GSil12A	0.014	0.200	0.098	0.105	0.277	9.670	0.290
GSil12B	0.022	0.263	0.046	0.056	0.346	7.396	0.183
After wash cycle No. 2							
GSil3A	0.000	0.076	0.053	0.049	0.084	2.895	0.178
GSil3B	0.000	0.014	0.015	0.015	0.027	0.804	0.045
GSil12A	0.000	0.039	0.043	0.041	0.057	2.620	0.125
GSil12B	0.006	0.033	0.015	0.021	0.053	2.464	0.048
After wash cycle No. 3							
GSil3A	0.000	0.066	0.054	0.050	0.087	3.081	0.198
GSil3B	0.000	0.022	0.015	0.015	0.030	0.939	0.062
GSil12AI	0.000	0.058	0.044	0.040	0.047	1.136	0.156
GSil12B	0.000	0.035	0.014	0.014	0.026	1.432	0.072
After wash cycle No. 4							
GSil3A	0.000	0.031	0.052	0.049	0.077	1.762	0.137
GSil3B	0.000	0.013	0.016	0.017	0.046	1.167	0.047
GSil12A	0.000	0.017	0.000	0.038	0.043	0.662	0.106
GSil12B	0.000	0.006	0.015	0.015	0.022	0.815	0.042

**Table 4:** Metal cations concentration (mg/L) after adsorption and desorption tests. The *Q* values for the adsorption test calculated as *Q* = $\frac{\left(C_{o}-C_{e}\right)V}{W}$ is also reported (see the Experimental section).

Sample	Zn^2+^	Cd^2+^	Pb^2+^	Cu^2+^
Metal cations concentration after adsorption test (mg/L)				
GSil3A	<lod *	<lod *	0.013	<lod *
GSil3B	0.028 ± 0.009	0.035 ± 0.008	0.017 ± 0.004	0.062 ± 0.008
GSil12A	0.007 ± 0.002	0.008 ± 0.001	0.015 ± 0.005	0.015 ± 0.005
GSil12B	0.019 ± 0.008	0.014 ± 0.007	0.027 ± 0.005	0.067 ± 0.008
Metals concentration after desorption test (mg/L)				
GSil3A	<lod	<lod	<lod	0.014 ± 0.008
GSil3B	<lod	<lod	0.055 ± 0.004	0.13 ± 0.01
GSil12A	0.114 ± 0.005	0.016 ± 0.002	0.209 ± 0.006	0.31 ± 0.02
GSil12B	<lod	<lod	0.021 ± 0.002	<lod
Q values for adsorption test (mg/g sample)				
GSil3A	0.383	0.383	0.383	0.383
GSil3B	0.120	0.120	0.120	0.120
GSil12A	0.341	0.341	0.341	0.341
GSil12B	0.111	0.111	0.111	0.110

* Limit of detection (lod) for Zn^2+^, Cd^2+^, Pb^2+^, Cu^2+^ are: 4.2 ng/L, 0.8 ng/L, 0.5 ng/L, 0.5 ng/L respectively.

**Table 5:** Compressive strength (σ_c_) and Young’s modulus (E) of GSil03 and GSil12 as determined before and after washing treatments.

Sample	σ_c_ (MPa)		E (MPa)	
	Before Washing	After Washing	Before Washing	After Washing
GSil03	10 ± 1	11 ± 1	800 ± 60	1100 ± 200
GSil12	2.2 ± 0.6	2.3 ± 0.8	300 ± 50	300 ± 50
